# MOK, a pharmacopuncture medicine, regulates thyroid dysfunction in L-thyroxin-induced hyperthyroidism in rats through the regulation of oxidation and the TRPV1 ion channel

**DOI:** 10.1186/s12906-017-2036-1

**Published:** 2017-12-15

**Authors:** Ji Hye Hwang, Seok Yong Kang, An Na Kang, Hyo Won Jung, Chul Jung, Jin-Ho Jeong, Yong-Ki Park

**Affiliations:** 1 Department of Acupuncture & Moxibustion Medicine, College of Korean Medicine, Gachon University, Seongnam, 13120 Republic of Korea; 2 Department of Herbology, College of Korean Medicine, Dongguk University, Gyeongju, 38066 Republic of Korea; 3 Namsangcheon Korean Medicine Clinic, Seoul, 06656 Republic of Korea; 4 Jisung-Kyunghee Korean Medicine Clinic, Seoul, 02803 Republic of Korea

**Keywords:** Antioxidation, Body temperature, Hyperthyroidism, L-Thyroxine, MOK, Pharmacopuncture, Thyroid hormones, TRPV1 channel

## Abstract

**Background:**

In this study, we evaluated the therapeutic effect of MOK, a pharmacopuncture medicine, on thyroid dysfunction in L-thyroxin (LT4)-induced hyperthyroidism rats.

**Methods:**

The experimental hyperthyroidism model was prepared by the intraperitoneal injection of LT4 (0.5 mg/kg) once daily for 2 weeks in SD rats. MOK extract was injected at doses of 0.3 or 3 mg/kg on acupuncture points in the thyroid glands of LT4-induced hypothyroidism rats once a day for 2 weeks. The body temperature, body weight, and food/water intake were measured once a week for 2 weeks. The levels of thyroid hormones, total cholesterol, LDL-cholesterol, GOT, and GPT were measured in the sera of rats using ELISA and an automatic blood analyzer. The histological changes of thyroid tissues were observed by H&E staining. The expression of thermo-regulating protein, TRPV1 was determined by western blot in dorsal root ganglion (DRG) and brain tissues. We also measured the contents of GSH in the liver and antioxidant enzymes, SOD, and catalase in the liver, heart, and brain tissues by enzyme-based assay and Western blot, respectively.

**Results:**

The acupuncture of MOK extract on the thyroid gland of LT4-induced hyperthyroidism rats significantly decreased the body temperature, and did not change body weight and food and water intakes. MOK acupuncture significantly increased the level of TSH, and decreased the levels of T3 and T4 in hyperthyroidism rats. The expression of TRPV1 was inhibited in both DRG and brain tissues after MOK acupuncture, and the levels of GOT, GPT, total cholesterol, and LDL-cholesterol were also decreased. MOK acupuncture also inhibited the pathological feature with follicular lining epithelial thicknesses and increased follicular colloid depositions in the thyroid glands of hypothyroidism. MOK acupuncture significantly increased hepatic GSH levels and decreased the expression of SOD and catalase in the liver, heart, and brain tissues of hyperthyroidism rats.

**Conclusions:**

These results suggest that the pharmacopuncture with MOK extract in hyperthyroidism can improve the pathophysiological changes through regulating the body temperature, thyroid hormones imbalance, lipid accumulation, and oxidation. This anti-hyperthyroidism effect of MOK pharmacopuncture is thought to be related to the control of thermo-regulating protein TRPV1 in DRG and brain.

## Background

Thyroid hormone plays an important role in development, metabolism, thermoregulation, and growth in the body. However, under the pathological conditions of several diseases such as Graves’ disease and tumors of the thyroid and pituitary glands, thyroid cells produce more hormones, causing a hyperthyroid state [1]. Hyperthyroidism leads to oxidative damage of the liver [2–4], osteoporosis [5], heart failure [6], and increased risk of heart attack [7]. It induces excessive production of thyroid hormones by the thyroid gland and includes the symptoms such as heat intolerance, fatigue or muscle weakness, mood swings, hand tremors, weight loss, and goiter, which is an enlarged thyroid. Graves’ disease, an autoimmune disorder, is the most common form of hyperthyroidism [8]. Although hyperthyroidism induces excessive synthesis and secretion of thyroid hormones from the thyroid gland, the pathophysiology is still unclear.

For the treatment of hyperthyroidism, radioactive iodine, anti-thyroid medication for the prevention of excessive thyroid hormone production and beta-blockers for controlling high blood pressure have been used on a long-term basis in modern medicine [9]. Unfortunately, these treatments occasionally induce negative side effects such as changing the symptoms into those of hypothyroidism [10], and use restriction of the propylthiouracil (PTU), which are increase a danger of agranulocytosis, systemic vasculitis, and renal failure in clinics [11]. However, hypothyroidism occurs with a fairly constant frequency for many years after therapy and may be unavoidable if cure of the disease is to be achieved by radioactive iodine [9]. Thus far, there is no fundamental cure, and treatment is limited to symptomatic therapy to correct the hyper-metabolic state with the fewest side effects and the lowest incidence of hypothyroidism.

The use of complementary and alternative medicine (CAM) in conjunction with conventional treatments in modern medicine may be safer and more effective. Traditional medicines, including Chinese medicine and Korean medicine, regard the treatment of both hyperthyroidism and hypothyroidism as a concept of Yin/Yang imbalance. When treating either condition, traditional medicines typically employ acupuncture, herbal medicines, and dietary therapeutics to rebalance an individual’s Yin and Yang. Thus, several experimental or clinical studies suggest that acupuncture and herbal medicines can be beneficial in treating hyperthyroidism [12]. Acupuncture is considered as the most common traditional therapy in Korea and China, as well as many other countries. Acupuncture is a method of stimulating acupuncture points which are the specific points on the body where a needle is inserted in the surface of the skin. Pharmacopuncture which is also called acupoint injection in traditional Chinese medicine (TCM) and traditional Korean medicine (TKM), is a new method of acupuncture in TCM and TKM involving an injection of herbal medicine at acupuncture points. This is considered to be faster and more effective than oral treatment because it can transfer the medicine to the target tissue/organ, directly without passing through the digestive system [13, 14]. Pharmacopuncture is commonly used in the clinical practice of TCM and TKM for the regulation of immune imbalance and the recovery of body homeostasis.

MOK is a polyherbal medicine including ten different constituents for pharmacopuncture therapy in Korean clinics. Pharmacopuncture with MOK is used for the treatment of patients with thyroid diseases such as hypothyroidism, hyperthyroidism, and heart issues. Although MOK is commonly used in Korean clinics and some studies have reported its anti-inflammatory and antioxidative effects [15], it has little scientific evidence. Therefore, in this study, we investigated the effects of the pharmacopuncture with MOK on thyroid dysfunction in L-thyroxin (LT4)-induced hyperthyroidism rats, and the possible mechanism.

## Methods

### Preparation of MOK extract

MOK was extracted with 10 herbs (Table 1), which were purchased from herbal materials company (Jayeondameun, Yangju, Korea) and authenticated by the Korean Food and Drug Administration (KFDA), in water and alcohol (*v*/v = 1:1) in a Good Manufacturing Practice (GMP)-compliant facility (Korea Immuno-Pharmacopuncture Association, Seoul, Korea), and was provided by the Korea Immuno-Pharmacopuncture Association (Seoul, Korea) as the finished product (53.1 mg/mL in a sealing vial). The quality of all herbs was approved by the Korea Food & Drug Administration (KFDA). For this study, the MOK extraction was dissolved in saline.

**Table 1:** Constituents of MOK extract

Herbal name	Scientific name	Ratio (mg/ml)
Hominis Placenta	*Hominis Placenta*	2
Moschus	*Moschus berezovskii*	0.5
Fel Ursi	*Ursus arctos*	0.3
Calculus Bovis Cow bezoar	*Bos taurus*	0.3
Scutellariae Radix	*Scutellaria baicalensis*	10
Phellodendri Cortex	*Phellodendron amurense*	10
Pulsatilla Koreana	*Pulsatilla koreana*	10
Sophorae Subprostratae Radix	*Sophora tonkinensis*	10
Aucklandiae Radix	*Aucklandia lappa*	5
*Aquilaria agallocha*	*Aquilaria agallocha*	5

### Experimental animals

Male Sprague-Dawley (SD) rats, aged 7 weeks, were purchased from SLC, Inc. (Shizuoka, Japan). All animals received food and water ad libitum and were housed under standard laboratory conditions at an ambient temperature of 22 ± 3 °C with humidity of 60 ± 5% under a daily 12 h/12 h light/dark schedule. All animals were handled according to the animal welfare guidelines issued by the Korean National Institute of Health and the Korean Academy of Medical Sciences for the care and use of laboratory animals. This study was conducted with the approval of the Institutional Animal Ethics Committee of Dongguk University (No. 130387).

### Induction of hyperthyroidism

**Fig. 1:**
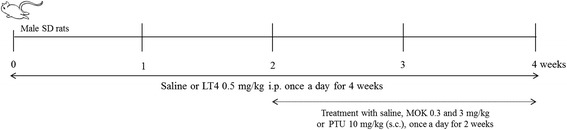
Experimental design in rat model of hyperthyroidism

Rats were randomly divided into five groups (*n* = 5): normal, hyperthyroidism-induced control, the MOK-pharmacopuncture (MOK group, 0.3 or 3 mg/kg, body weight; bw) group in hyperthyroidism rats, and the 6-Propyl-2-thiouracil (PTU, Sigma-Aldrich, CA, USA) pharmacopuncture (PTU group, 10 mg/kg, bw) group in hypothyroidism rats as a reference group. Hyperthyroidism was induced by intraperitoneal injection of LT4 (0.5 mg/kg, bw). In normal rats, only saline was intraperitoneally injected (Fig. 1). After 14 days, the rats were administrated MOK pharmacopuncture into the anterior neck near the thyroid gland at a volume of 0.05 mL/rat, dissolved in saline, once daily from days 15 to 28 in LT4-induced hyperthyroidism rats. As a reference drug, PTU (6-Propyl-2-thiouracil: Sigma-Aldrich, CA, USA) 10 mg/kg (body weight) was dissolved in 0.3 ml saline, and rats were given a daily subcutaneous injection of PTU into the dorsal neck.

### Measurement of body and organ weights, food and water intake, and body temperature

The body weight in each group, and food and water intake were measured at the initiation of treatment and once a week for 4 weeks. The weights of the heart, liver, and spleen were also measured at the final day, and their relative weights were calculated as the percentage of body weight. The body temperature was measured once a week using a thermometer (Thermalert TH-8, Physitemp Instruments, Clifton, NJ, USA) with a rectal probe. The probe was inserted 3 cm into the rectum while the rat was gently restrained. A steady readout was obtained within 30 s of probe insertion.

### Measurement of serological parameters

Blood samples were collected by cardiac puncture under 2.5% isoflurane anesthesia, sacrificing the rats on day 29. Blood was clotted for 2 h at room temperature and centrifuged at 5000 rpm for 10 min at 4 °C to obtain serum. The concentration of thyroid hormones (TSH, T3, and T4) was measured in the sera of all animals using commercially available enzyme-linked immunosorbent assay (ELISA) kits according to the manufacturer’s recommendations (Cusabio, China). The concentration of each hormone was calculated from the standard curve for each recombinant protein in the ELISA kits. The levels of glutamyloxaloacetic transaminase (GOT), glutamyl pyruvic transaminase (GPT), total cholesterol, high-density lipoprotein (HDL)-cholesterol, low-density lipoprotein (LDL)-cholesterol, triglyceride (TG), and glucose were measured in the sera by an automated blood analyzer (FDC7000i, Fujifilm Co., Japan).

### Histological analysis

On day 29, all rats were sacrificed, and thyroid tissues were isolated for histological examination. The tissues were fixed in 4% paraformaldehyde solution, embedded in paraffin, and longitudinally cut into 5-μm serial sections. The sections were then stained with H&E to assess morphological changes of the thyroid glands. The size of thyroid follicles was also calculated using ImageJ (National Institutes of Health, Bethesda, MD, USA) in H&E stained tissues.

### Western blot analysis

To investigate the effects of MOK pharmacopuncture on the oxidation of liver, heart, and brain tissues, as well as the expressions of transient potential vanilloid 1 (TRPV1) protein in DRG and brain tissues, we conducted western blot analysis. Briefly, livers, hearts, brains, and DRG tissues were harvested from each group, minced, and homogenized with an electric homogenizer in 5 volumes of extraction buffer (100 mM Tris, pH 7.4, 150 mM sodium chloride (NaCl), 1 m Methylene glycol-bis(β-aminoethyl ether)-N,N,N′,N′-tetraacetic acid (EGTA), 1 mMethylenediaminetetraacetic acid (EDTA), 1% Triton X-100, and 0.5% sodium deoxycholate). The tissue lysates were placed on a shaker at 4oC for 1 h and centrifuged at 10,000 xg for 5 min. Protein concentrations were determined by the Bradford assay (Bio-Rad, Hemel, Hempstead, UK). A total of 30 μg/mL of protein was separated on a 10 to 12% sodium dodecyl sulfate (SDS)-polyacrylamide gel and then transferred to a nitrocellulose membrane (Millipore, Billerica, MA, USA). Each membrane was incubated for 1 h with 5% skim milk in TBS-T buffer (0.1 M Tris-HCl, pH 7.4, 0.9% NaCl, 0.1% Tween-20) to block non-specific binding and incubated with primary anti-SOD2, CAT, and TRPV1 (Cell Signaling Technology, Inc.), and β-actin (Sigma-Aldrich) antibodies. The membranes were incubated with peroxidase-conjugated affinity goat anti-rabbit IgG (Santa Cruz Biotech). Each protein was detected using a chemiluminescence detection system according to the manufacturer’s instructions (ECL, Amersham, Berkshire, UK). The band intensity was quantified by densitometric analysis using ImageJ software (NIH, USA).

### Measurement of total glutathione levels

The contents of total glutathione was measured in the sera of all animals using the GSH/GSSG assay kit (Cell Biolabs, Inc., USA) based on the presence of glutathione reductase that reduces GSSG to GSH in the presence of nicotinamide adenine dinucleotide phosphate-oxidase (NADPH). Subsequently, the chromogen reacts with the thiol group of GSH to produce a colored compound that absorbs at 405 nm). Data were expressed as μmol of GSH per gram of liver tissue.

### HPLC analysis

To identify the constituents of the MOK extract, high-performance liquid chromatography (HPLC) was conducted using standard compounds. The HPLC apparatus was a WatersDelta600 (Waters, Milford, MA, USA) with PDA (Photodiode Array) Detector. A CAPCELL PAK C18 UG80 column (SHISHEIDO Co., Ltd. Japan) was employed. Chromatographic separation was performed using a gradient solvent system consisting of acetonitrile with 0.1% formic acid (B) and water (A). The gradient program was as follows: 0 min, 0%B; 5 min, 3%B; 15 min, 3%B; 25 min, 10%B; 35 min, 15%B; 45 min, 15%B; 55 min, 30%B; 65 min, 50%B; 75 min, 70%B; and 80 min, 0%B. The column eluent was monitored at UV 254 nm. All solvents were subsequently degassed with 0.2 μm cellulose acetate filtering. Chromatography was performed at room temperature with a flow rate of 1.0 mL/min, and a 25 μL volume was analyzed.

### Statistical analysis

Data were expressed as the mean ± SD (standard deviation) of each group (*n* = 5 per group), and all the data were analyzed GraphPad Prism (Prism 5.01, GraphPad Software, La Jolla, CA, USA). The statistical significances were analyzed using one-way analysis of variance (ANOVA) to compare the groups, followed by Dunnett’s test. Null hypotheses of no difference were rejected if *p*-values were less than 0.05.

## Results

### Effect of MOK pharmacopuncture on the changes of body weights and food and water intakes in hyperthyroidism rats

**Fig. 2:**
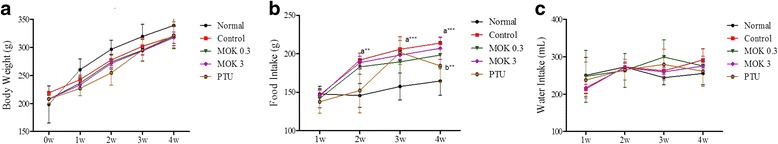
Effects of MOK pharmacopuncture on the changes of physiological parameters in LT4-induced hyperthyroidism rats. MOK pharmacopuncture was subcutaneously administered once daily for 2 weeks, and measured the body weight (**a**), and food and (**b**) and water (**b**) intake once a week. Data are presented as mean±SD (*n*=5 per each group). *P<0.05, **P<0.01 and ***P<0.001 vs normal (**a**) or control (**b**). Normal, normal group; Control, LT4-induced hyperthyroidism group; MOK 0.3, MOK 0.3 mg/kg-treated group in control; MOK 3, MOK 3 mg/kg-treated group in control; and PTU, PTU 10 mg/kg-treated group as a reference drug

To investigate the effects of MOK pharmacopuncture on the changes in physiological parameters, we measured the body weights and food/water intakes once a week. As a result, the body weight was increased in the LT4-induced hyperthyroidism group compared with the normal group, and the treatment of MOK-pharmacopuncture at 0.3 and 3 mg/kg was not effective in the decrease of body weights in hyperthyroidism rats (Fig. 2a). The food (Fig. 2b) and water (Fig. 2c) consumption was also increased in hyperthyroidism rats, and these increases were not changed by the treatment of MOK pharmacopuncture.

**Fig. 3:**
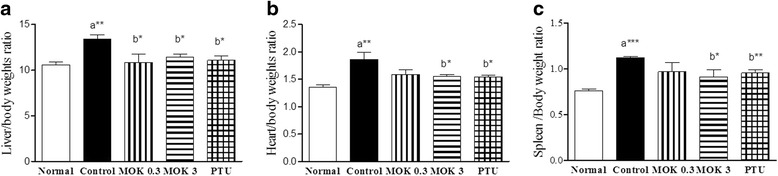
Effects of MOK pharmacopuncture on the changes of organ weights in LT4-induced hyperthyroidism rats. MOK pharmacopuncture was subcutaneously administered once daily for 2 weeks, and the weights of liver (**a**), heart (**b**), and spleen (**c**) were also measured in LT4-induced hyperthyroidism rats. Relative organ weights to body weights were measured. Data are presented as mean±SD (*n*=5 per each group). *P<0.05, **P<0.01 and ***P<0.001 vs normal (**a**) or control (**b**). Normal, normal group; Control, LT4-induced hyperthyroidism group; MOK 0.3, MOK 0.3 mg/kg-treated group in control; MOK 3, MOK 3 mg/kg-treated group in control; and PTU, PTU 10 mg/kg-treated group as a reference drug

Next, we measured the weights of livers, hearts, and spleens in all groups. The weights of livers, hearts, and spleens were significantly increased in hyperthyroidism rats compared with rats in the normal group. These increases were significantly decreased in the MOK pharmacopuncture-treated group compared with the control group (Fig. 3), indicating that MOK pharmacopuncture has no effect on the changes of body weight, and food/water intake in hyperthyroidism rats, but it prevents organ loss from hyperthyroidism-induced damage.

### Effect of MOK pharmacopuncture on body temperature and the expression of the TRPV1 channel in hyperthyroidism rats

**Fig. 4:**
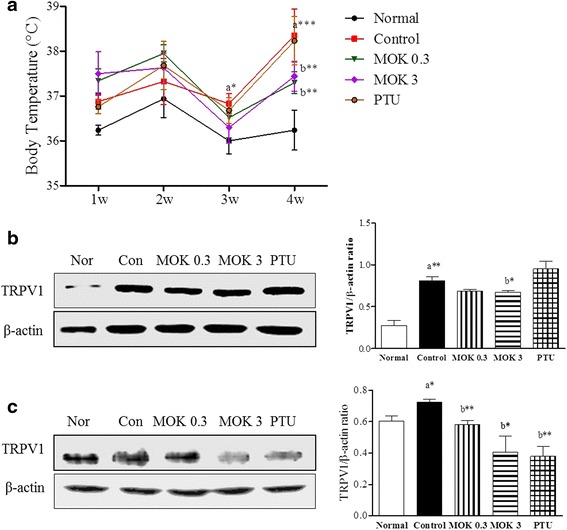
Effect of MOK pharmacopuncture on the changes pharmacopuncture on the changes of body temperature and the expression of TRPV1 protein in LT4-induced hyperthyroidism rats. MOK pharmacopuncture was subcutaneously administered once daily for 2 weeks and the body temperature was measured by rectal thermometer (**a**) once a week. The production of TRPV1 protein was determined in DRG (**b**) and brain (**c**) tissues isolated from LT4-induced hyperthyroidism rats using Western blot. Data are presented as mean ± SD (*n* = 5 per each group). *P < 0.05, **P < 0.01 and ***P < 0.001 vs normal (**a**) or control (**b**). Normal, normal group; Control, LT4-induced hyperthyroidism group; MOK 0.3, MOK 0.3 mg/kg-treated group in control; MOK 3, MOK 3 mg/kg-treated group in control; and PTU, PTU 10 mg/kg-treated group as a reference drug

To investigate the effects of MOK pharmacopuncture on the regulation of body temperature, we measured the changes of body temperature, and the thermo-regulator TRPV1 proteins in DRG and brain tissues using western blot. The body temperature was significantly increased in the LT4-induced hyperthyroidism control group from 2 and 4 weeks after LT-4 injection, and this increase was significantly decreased by the treatment of MOK pharmacopuncture at 0.3 and 3 mg/kg (Fig. 4a). In the PTU-treated group, the body temperature was lower than that of the hyperthyroidism group.

In the expression of TRPV1, the treatment of MOK pharmacopuncture in hyperthyroidism was significantly decreased in both DRG (Fig. 4b) and brain (Fig. 4c) tissues in a dose-dependent manner. The PTU treatment also inhibited the TRPV1 expression in the DRG tissues, but did not affect brain tissues. These results indicate that MOK pharmacopuncture can help to decrease the body temperature through downregulation of the thermo-regulator TRPV1 expression in hyperthyroidism rats.

### Effect of MOK pharmacopuncture on the levels of thyroid hormones in hyperthyroidism rats

To investigate the effects of MOK pharmacopuncture on the production of thyroid hormones, we measured the levels of TSH, T4, and T3 in the sera of LT4-induced hyperthyroidism rats using ELISA.

**Fig. 5:**
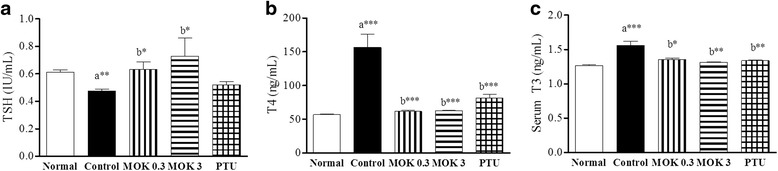
Effects of MOK pharmacopuncture on the levels of thyroid hormones in LT4-induced hyperthyroidism rats. MOK pharmacopuncture was subcutaneously administered once daily for 2 weeks and measured the levels of TSH (**a**), T4 (**b**), and T3 (**b**) in the sera of rats by ELISA, respectively. Data are presented as mean ± SD (*n*=5 per each group). *P<0.05, **P<0.01 and ***P<0.001 vs normal (**a**) or control (**b**). Normal, normal group; Control, LT4-induced hyperthyroidism group; MOK 0.3, MOK 0.3 mg/kg-treated group in control; MOK 3, MOK 3 mg/kg-treated group in control; and PTU, PTU 10 mg/kg-treated group as a reference drug

As shown in Fig. 5a, the TSH levels were significantly decreased in the hyperthyroidism group compared to the normal group. MOK pharmacopuncture was found to be significantly increased the TSH levels at 0.3 and 3 mg/kg in hyperthyroidism rats, similar to the PTU-treated group. The levels of T4 (Fig. 5b) and T3 (Fig. 5c) were significantly increased in the hyperthyroidism group, but these were significantly decreased by the treatment of MOK pharmacopuncture. These results indicate that MOK pharmacopuncture can improve the symptoms of hyperthyroidism, such as heat intolerance, through the regulation of thyroid hormone imbalance.

### Effects of MOK pharmacopuncture on histopathological changes of thyroid glands in hyperthyroidism rats

**Fig. 6:**
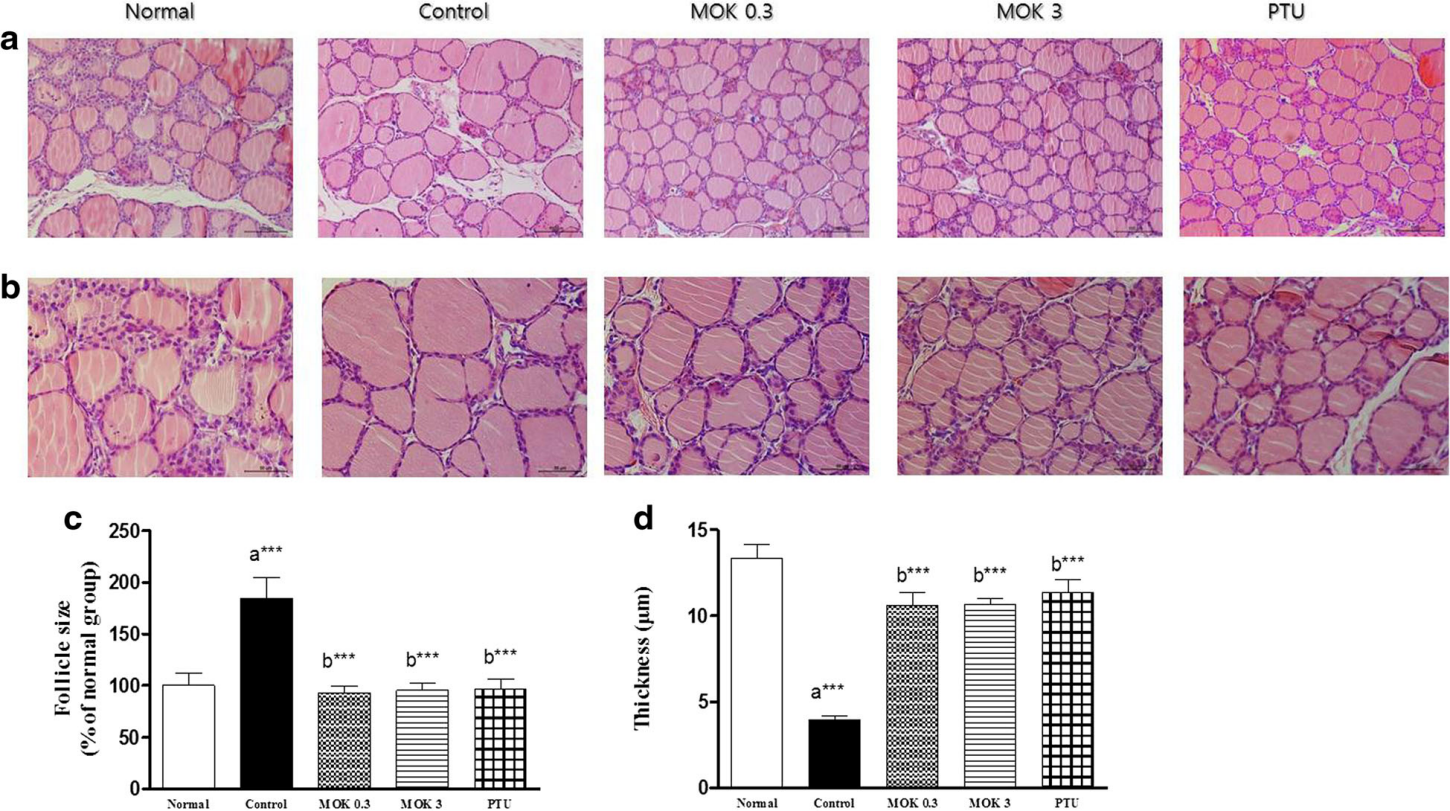
Effects of MOK pharmacopuncture on the histopathological changes of thyroid tissues in LT4-induced hyperthyroidism rats. MOK pharmacopuncture was subcutaneously administration once daily for 2 weeks, and isolated thyroid glands from the rats. Thyroid tissues were prepared paraffin-formatted slide, and stained with H&E dye. Morphological changes were observed by microscope ×200 (**a**), and ×400 (**b**) in original magnification. The mean of relative follicular sizes to normalgroup (**c**) and the follicular lining epithelium thickness (**d**) were measured in LT4-induced hyperthyroidism rat.Data are presented as mean ± SD (*n* = 5 per each group). ^*^ *P* < 0.05, ^**^ *P* < 0.01 and ^***^ *P* < 0.001 vs normal (**a**) or control (**b**). Normal, normal group; Control, LT4-induced hyperthyroidism group; MOK 0.3, MOK 0.3 mg/kg-treated group in control; MOK 3, MOK 3 mg/kg-treated group in control; and PTU, PTU 10 mg/kg-treated group as a reference drug

Next, we investigated the effects of MOK pharmacopuncture on the histopathological changes of thyroid tissues using H&E staining. As a result, marked and noticeable atrophic changes of thyroid glands, decreases of total thyroid glands and the thickness of follicular lining epithelium, and increased follicular sizes in the LT4-induced hyperthyroidism group compared with the normal group (Fig. 6). However, these histopathological changes were inhibited by the treatment of MOK pharmacopuncture at 0.3 and 3 mg/kg, similar to the PTU-treated group. In particular, the follicular sizes in the LT4-induced hyperthyroidism group were significantly increased (185.7 ± 41.02%) compared to those of the normal group, but significantly decreased (49.6 ± 18.83% and 48.2 ± 21.62%) in MOK pharmacopuncture at 0.3 and 1.5 mg/kg and also significantly decreased (47.3 ± 27.07%) in the PTU-treated group (Fig. 6c). The thickness of the follicular lining of the epithelium was also significantly reduced in the LT4-induced hyperthyroidism group, but it was significantly increased after the MOK pharmacopuncture or PTU treatment (Fig. 6d).

### Effect of MOK pharmacopuncture on the serological changes in hyperthyroidism rats

**Fig. 7:**
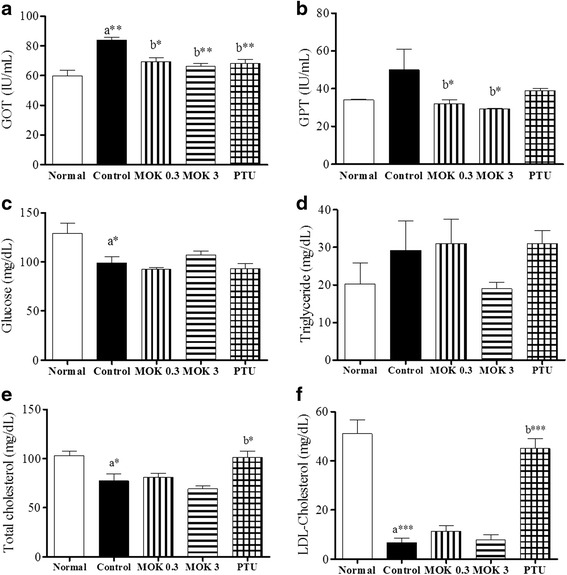
Effects of MOK pharmacopuncture on the changes of serological parameters in LT4-induced hyperthyroidism rats. MOK pharmacopuncture was subcutaneously administration once daily for 2 weeks, and measured the levels of GOT (**a**), GPT (**b**), glucose (**c**), triglyceride (**d**), total-cholesterol (**e**), and LDL-cholesterol (**f**) in the sera of rats by automatic blood biochemical analyzer. Data are presented as mean ± SD (*n* = 5 per each group). ^*^ *P* < 0.05, ^**^ *P* < 0.01 and ^***^ *P* < 0.001 vs normal (**a**) or control (**b**). Normal, normal group; Control, LT4-induced hyperthyroidism group; MOK 0.3, MOK 0.3 mg/kg-treated group in control; MOK 3, MOK 3 mg/kg-treated group in control; and PTU, PTU 10 mg/kg-treated group as a reference drug

To investigate the effects of MOK pharmacopuncture on the serological changes in hyperthyroidism, we measured the levels of GOT, GPT, glucose, triglycerides, total cholesterol, and LDL-cholesterol in the sera of all groups using an automatic blood analyzer. As shown in Fig. 6, serum levels of GOT (Fig. 7a) and GPT (Fig. 7b) as liver toxic markers were increased in the LT4-induced hyperthyroidism group, but these increases were significantly decreased by the treatment of MOK pharmacopuncture at 0.3, and 3 mg/kg similar to the PTU-treated group. However, the MOK pharmacopuncture did not change the levels of glucose and lipid accumulation (Fig. 7c, d, e, f).

### Effect of MOK pharmacopuncture on oxidation in liver, brain, and heart tissues in hyperthyroidism rats

**Fig. 8:**
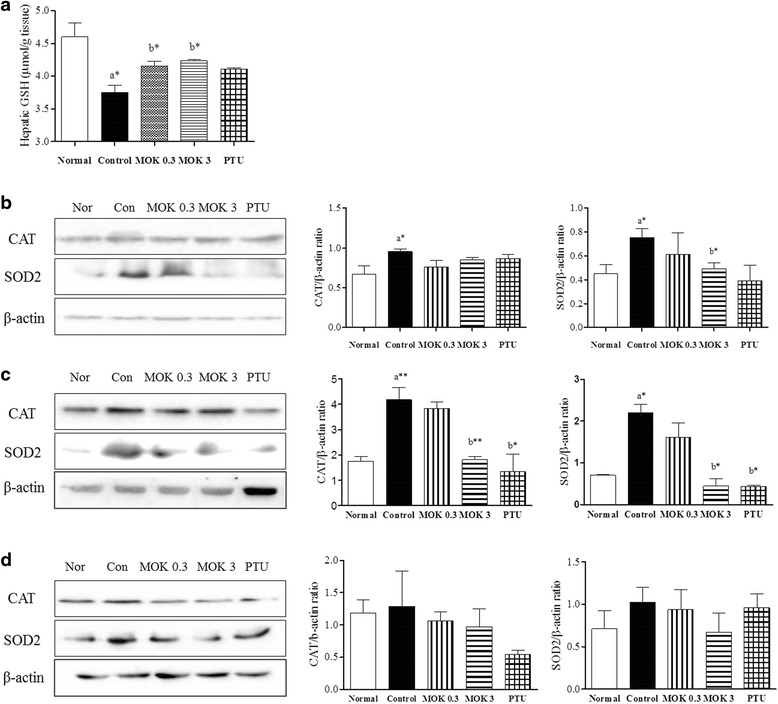
Effect of MOK pharmacopuncture on the oxidation in liver, brain, and heart tissues ofLT4-induced hyperthyroidism rats. MOK pharmacopuncture was subcutaneously administration once daily for 2 weeks, and measured the levels of GSH (**a**) from the liver of rats by ELISA. Theexpression of catalase (CAT) and SOD2in the liver (**b**), brain (**c**), and heart (**d**) tissues using Western blot. Data are presented as mean ± SD (*n* = 5 per each group). ^*^ *P* < 0.05, ^**^ *P* < 0.01 and ^***^ *P* < 0.001 vs normal (**a**) or control (**b**). Normal, normal group; Control, LT4-induced hyperthyroidism group; MOK 0.3, MOK 0.3 mg/kg-treated group in control; MOK 3, MOK 3 mg/kg-treated group in control; and PTU, PTU 10 mg/kg-treated group as a reference drug

To investigate the effect of MOK pharmacopuncture on oxidative damage in the body, we measured the levels of antioxidant substance GSH in the liver tissues of hyperthyroidism rats and also, the expression of antioxidant enzymes CAT and SOD2 in liver, brain, and heart tissues using enzyme-based assay and Western blot, respectively. The level of GSH was significantly reduced in the LT4-induced hyperthyroidism group, and it was significantly increased in the treatment of MOK pharmacopuncture at 0.3 and 3 mg/kg (Fig. 8a). Next, the expression of CAT was increased in the hyperthyroidism group, but this was decreased by treatment with MOK pharmacopuncture in the liver (Fig. 8b), brain (Fig 8c), and heart (Fig. 8d) of hyperthyroidism rats. SOD2 expression was also significantly increased in hyperthyroidism rats, but it was significantly decreased in liver and brain tissues by the treatment of MOK pharmacopuncture at 3 mg/kg. These results indicate that the treatment of MOK pharmacopuncture in hyperthyroidism rats can protect liver and brain tissues against oxidative damages.

### HPLC analysis

**Fig. 9:**
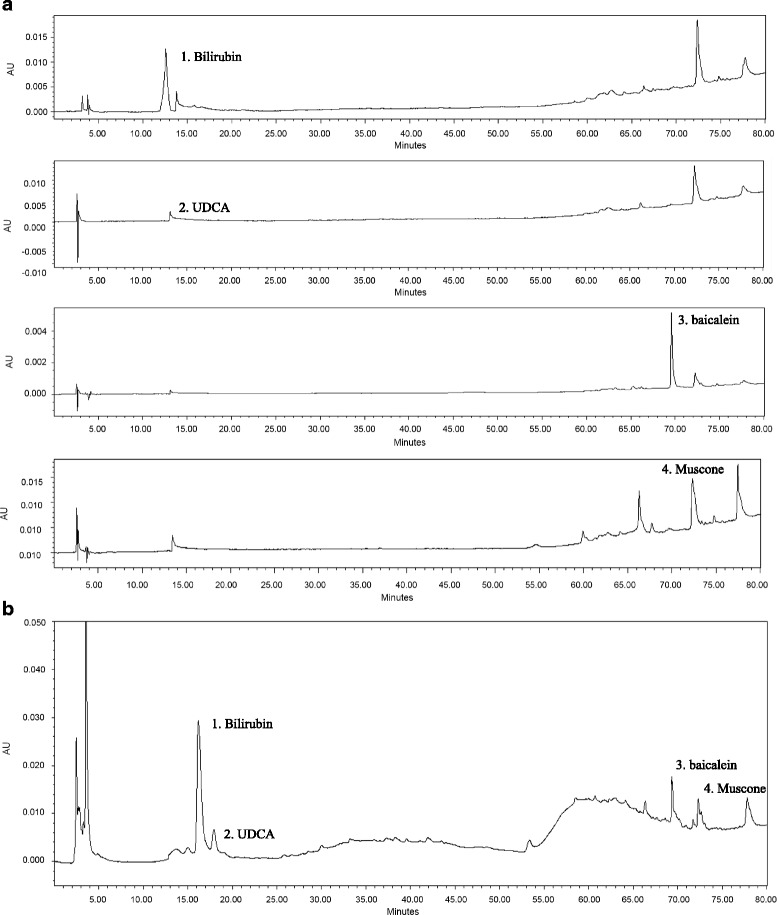
HPLC pattern analysis of the MOK extract: (**a**) each standard compound. Peaks: (1) bilirubin, (2) UDCA, (3) baicalein, and (4) muscone; and (**b**) MOK extract

In HPLC analysis of MOK extract, four compounds such as 3-methylcyclopentadecanone (muscone), ursodeoxycholic acid (UDCA), bilirubin, and 5,6,7-trihydroxyflavone (baicalein) in the MOK extract were identified through the comparison of retention times of authentic standards (Fig. 9).

## Discussion

Hyperthyroidism is often referred to as an overactive thyroid and it is caused by increases of thyroid hormones, such as TSH, and when the thyroid gland produces and secretes immoderate amounts of the free thyroid hormones, including T3 and T4, which are circulating in the blood [16, 17]. The abnormal levels of these hormones cause some biochemical and clinical abnormalities, especially in pathological states such as Graves’ disease and thyroid tumors, in which the pituitary gland stimulates thyroid cells, producing more hormones, resulting in a hyperthyroid state [8]. The hyperthyroid state is accompanied by an augmentation of the pro-oxidant to antioxidants ratio that elevates the accumulation of molecules as oxidative damage [2, 3], that induces secondary disease conditions, such as liver toxicity, osteoporosis [5], and heart failure [6]. Currently, there are no complete therapies for hyperthyroidism regardless of the cause, except for some alternative therapies, including anti-thyroid drugs, iodine-based radiotherapy, and thyroidectomy [18, 19].

In traditional medicines, such as TCM and TKM, acupuncture, herbal medicines, and dietary therapeutics have been used for maintenance of thyroid function in various thyroid disorders based on the therapeutic theory of rebalancing an individual’s Yin and Yang. Acupuncture is recognized by the World Health Organization (WHO) as a useful therapeutic method in thyroid diseases, but it is not frequently listed as a treatment for thyroid disorders, and a little evidence has appeared in the literature. Therefore, more scientific evidence from in vitro and in vivo studies, and clinical studies are needed.

Pharmacopuncture (acupoint injection) is a method of acupuncture that is frequently used in clinical practice for the treatment of thyroid diseases. Meanwhile, immuno-pharmacopuncture therapy, first developed by Nam Sang-cheon, has been used for the regulation of immune balance, in TKM clinics [13, 14]. MOK is a polyherbal medicine for the immuno-pharmacopuncture therapy and it is clinically used to treat the meridian of fire in nature and symptoms related to heart disease, such as fright palpitations, fearful throbbing, sleep disorders, angina pectoris, and thyroid diseases, such as hyperthyroidism, hypothyroidism, thyroid hypertrophy, and benign thyroid nodules [14]. Recently, the anti-inflammatory and antioxidative effects of MOK have been reported [15], but little scientific evidence of MOK has been found. Therefore, we investigated the therapeutic effects of MOK pharmacopuncture in an LT4-induced hyperthyroidism rat model.

The hyperthyroidism was easily achieved in rodents by continuous treatment of LT4, a synthetic form of the thyroid hormone [20]. The daily treatment of LT4 induces marked elevations of T3 and T4 levels and decreases in TSH levels, and also can cause oxidative stress relating to liver and heart damage, atrophic changes of the thyroid gland, and decreases in body fat mass [21]. In this study, we prepared an experimental hyperthyroidism rat by LT4 injection, and the anti-hyperthyroidism effects of MOK pharmacopuncture were investigated compared with PTU, as an antithyroidic drug [21, 22]. PTU, a thioamide drug, is commonly used to treat hyperthyroidism and decreases the amount of thyroid hormone from the thyroid gland by repressing the 5′-deiodinase, which converts T4 to the active form T3 [23]. Therefore, PTU has been selected as a reference drug for developing a new medicine to treat hyperthyroidism.

In our study, the treatment of MOK pharmacopuncture in LT4-induced hyperthyroidism rats significantly decreased the body temperature, prevented the weight loss of liver, heart, and spleen, regulated the levels of thyroid hormones TSH, T4, and T3, and also decreased the serum levels of GOT, and GPT similarly to the PTU-treated group. In previous studies, LT4-induced hyperthyroid rats were accompanied by a higher rectal temperature,^3^ and treatment with PTU induced a lower rectal temperature [24]. Our results indicate that MOK pharmacopuncture regulates heat intolerance in hyperthyroidism through decreasing the body temperature with downregulation of the thermo-regulator TRPV1 expression in DRG and brain tissues. Our sensory nerves use specialized ion-channel proteins to report environmental temperatures, most notably, but not exclusively, the TRP ion channels [25–27]. The TRPV1 channels in sensory nerves respond to heat and to capsaicin, an alkaloid from “hot” peppers, which binds to the open channel and thus depolarizes the neuron and fires action potentials [28]. The brain, interpreting this information as an increase in ambient temperature, initiates vasodilation and sweating. Conversely, drugs that block TRPV1 input to the brain provoke hypothalamic-mediated changes in metabolism that elevate body temperature [29]. In our study, MOK pharmacopuncture exhibited a thermoregulatory property through downregulation of the TRPV1 expression in DRG and brain tissues of LT4-induced hyperthyroidism rats. By contrast, PTU treatment did not show a significant decrease of TRPV1 expression in DRG, but was effective in the brain. These results suggest that MOK pharmacopuncture is more useful in controlling the body temperature than PTU in hyperthyroidism.

The laboratory evaluation of thyroid hormones, such as TSH, T3, and T4, in the blood is considered the best screening test for hyperthyroidism. The excessive production of these catabolic thyroid hormones is closely related with the progression of hyperthyroidism, and changes the basic body metabolisms and induces the depletion of deposited nutrients in cells, especially adipocytes and body weight decreases [30]. In our study, a notable decrease of TSH with an increase of T3 and T4 levels was detected in the sera of LT4-induced hyperthyroidism rats, and MOK pharmacopuncture increased TSH levels and coincidentally decreased the T3 and T4 levels, similar to PTU-treated rats. These results are considered direct evidence that MOK pharmacopuncture can partially control LT4-induced imbalance of thyroid hormones.

The marked size decrease of the thyroid gland and its atrophic changes at gross inspections were observed in hyperthyroidism. The LT4 treatment in rats also showed decreases of thyroid gland thickness and the follicular lining epithelium, and increases of follicular diameters that were related to deposition of colloids in the lumens [31]. In our study, histopathological atrophic changes of thyroid glands with the follicular lining epithelial thicknesses and increased follicular size by colloid depositions were also observed in LT4-induced hyperthyroidism rats. These changes were inhibited by MOK pharmacopuncture or PTU treatment.

Higher relative weights of organs, such as the liver, heart, spleen, and pancreas have been reported in LT4-induced hyperthyroidism animal models [32]. In our study, the injection of LT4 significantly increased the organ weights of the liver, heart, and spleen in rats, and MOK pharmacopuncture significantly decreased their relative weights. The liver is an important target organ for thyroid hormones with crucial biological and medical implications [33], and severe damage accompanies the imbalances of thyroid hormones in thyroid dysfunction [2, 3].Clinical diagnosis of thyroid diseases and damage to the structural integrity of the liver is commonly evaluated by monitoring the status of serum GOT and GPT activities [34]. Additionally, higher activities of these hepatic enzymes are found in reaction to hyperthyroidism-induced oxidative stress [3, 4]. Thus, thyroid hormones are known to play an important role in hepatocyte proliferation and hepatic hyperplasia in the sinusoidal space [3, 16]. In our study, the serum levels of GOT and GPT were increased in LT4-induced hyperthyroidism rats, and this increase was decreased by MOK pharmacopuncture, suggesting that MOK pharmacopuncture has a hepatoprotective effect in hyperthyroidism.

Hyperthyroidism is also closely related with the development of hypocholesterolemia or unexplained improvement of the lipid profile by excessive production of thyroid hormones. It has been reported that patients with hyperthyroidism are shown lower serum levels of VLDL-, LDL-, and HDL-cholesterols, apolipoprotein B (apoB), lipoprotein-α (Lpα) and proproteinconvertasesubtilisin/kexin type 9 (PCSK9) [35]. In our study, LT4 treatment decreased the levels of total cholesterol and LDL-cholesterol and increased TG levels, while this was recovered with MOK pharmacopuncture, suggesting that MOK pharmacopuncture can ameliorate the abnormal lipid metabolism in hyperthyroidism.

Hyperthyroidism leads to an enhancement of the metabolic rate and, more specifically, of the oxidative metabolism [36]. GSH is one of the most significant antioxidants involved in cellular protection against oxidative stress, and its reduction has the ability to give the unstable molecules, such as the reactive oxygen species (ROS), the reducing equivalents. In the condition of reduced cellular GSH levels and GSH synthesis capacity, many cells, tissues, and organs become susceptible to oxidative stress. The activity increase of several antioxidant enzymes, such as SOD and CAT, may be indicative of the failure to compensate for the induced oxidative stress.^4^ In hyperthyroidism, the increase of SOD and CAT activities indicates that prevention of ROS overproduction fails to compensate for the induced oxidative stress [37, 38]. Therefore, increasing interest is being shown in the use of antioxidants as therapeutic agents to ameliorate elevated oxidative stress in various diseases and pathophysiological disorders [3, 8]. In our study, the treatment of MOK pharmacopuncture in hyperthyroidism rats significantly increased the expression of SOD and CAT in liver and brain tissues, suggesting that MOK pharmacopuncture is useful to treat hyperthyroidism through the protection of the liver and brain against oxidative stress-induced damage.

HPLC analysis revealed that MOK extract contained the compounds such as bilirubin, UDCA, baicalein, and muscone. Bilirubin is the main compound of *Bos Taurus* and was reported to have antiinflammation, anticancer and antiviral activities [39]. UDCA, a secondary bile acid, is the main compound of *Ursus arctos* and was also reported anticancer [40], antioxidation, and liver protective effects [41]. Baicalein is the main flavonoid of *Scutellaria baicalensis* and was reported the antioxidant [42], anticancer and antiestrogenic effects [43]. Muscone is the standard compound of *Moschus berezovskii* and has been reported to have the effects of neuroprotection, antioxidation [44], antiinflammation [45], and alleviation of pain and cardiovascular dysfuntion [46]. In our HPLC analysis, these compounds might be involved in the therapeutic effects of MOK on hyperthyroidism, however, their function responsible for MOK pharmacopuncture remains a problem for further study.

## Conclusions

MOK pharmacopuncture treatment in LT4-induced hyperthyroidism rats improved the pathophysiological features with increases in body temperature, regulation of thyroid hormones and lipid metabolism, and inhibition of thyroid gland, liver, and brain damage through regulation of the TRPV1 channel and antioxidation. These results suggest that MOK pharmacopuncture can be a useful acupuncture therapy for the treatment of patients with hyperthyroidism.

## Abbreviations

BW: Body Weight
CAM: Complementary and Alternative Medicine
DRG: Dorsal Root Ganglion
GOT: Glutamyl Oxaloacetic Transaminase
GPT: Glutamyl Pyruvic Transaminase
GSH: Glutathione
HDL: High-Density Lipoprotein
KFDA: Korean Food and Drug Administration
LDL: Low-Density Lipoprotein
LT4: L-thyroxin
PTU: Propylthiouracil
T3: Triiodothyronine
T4: Tetraiodothyronine
TCM: Traditional Chinese Medicine
TG: Triglyceride
TKM: Traditional Korean Medicine
TRPV1: Transient Potential Vanilloid 1
TSH: Thyroid Stimulating Hormone
UDCA: Ursodeoxycholic Acid
